# Long Term Outcome of Routine Image-enhanced Endoscopy in Newly Diagnosed Head and Neck Cancer: a Prospective Study of 145 Patients

**DOI:** 10.1038/srep29573

**Published:** 2016-07-08

**Authors:** Chen-Shuan Chung, Wu-Chia Lo, Ming-Hsun Wen, Chen-Hsi Hsieh, Yu-Chin Lin, Li-Jen Liao

**Affiliations:** 1 Departments of Internal Medicine, Far Eastern Memorial Hospital, New Taipei City, Taiwan; 2 College of Medicine, Far Eastern Memorial Hospital, Fu Jen Catholic University, New Taipei City, Taiwan; 3 Departments of Otolaryngology, Far Eastern Memorial Hospital, New Taipei City, Taiwan; 4 Departments of Radiation Oncology, Far Eastern Memorial Hospital, New Taipei City, Taiwan; 5 Department of Medicine, Far Eastern Memorial Hospital, School of Medicine, National Yang-Ming University, Taipei, Taiwan; 6 Departments of Medical Oncology and Hematology, Far Eastern Memorial Hospital, New Taipei City, Taiwan; 7 Department of Electrical Engineering, Yuan Ze University, Taoyuan, Taiwan

## Abstract

Synchronous second primary tumors (SPTs), especially esophageal squamous cell neoplasia (ESCN), in patients with head and neck squamous cell carcinoma (HNSCC) are not uncommon. Image-enhanced endoscopy (IEE) screening may identify SPTs while there is no evidence to support its benefit. We prospectively recruited an adult cohort with newly-diagnosed HNSCC for IEE screening of upper gastrointestinal (UGI) tract neoplasia. 145 HNSCC patients were recruited. 22 (15.2%) patients had synchronous UGI tract neoplasia, including 20 ESCNs and 2 gastric adenocarcinoma. At a median follow-up of 2.72 (±1.73) years, the 3-year overall survival (OS) rate was 0.71. HNSCC patients with synchronous ESCN/UGI tract neoplasia had poorer prognosis than those without (multivariate analysis, hazard ratio [HR] 2.75/2.79, 95% confidence interval [CI] 1.11~6.82/1.15~6.80, p = 0.03/0.02). HNSCC patients with advanced (stage III&IV) ESCN had worst survivals (p < 0.001). Among those with synchronous ESCNs, hypopharyngeal cancers were associated with poorer prognosis when compared with oral cancers (HR 2.36, 95% CI 1.08~5.15, p = 0.03). IEE screening for UGI SPTs in HNSCC patients could be used for risk stratification and prognosis prediction. HNSCC patients with advanced ESCN had the worst prognosis. Further studies are needed to demonstrate the survival benefits from IEE screening.

Because of “field cancerization”, mucosa of the upper digestive tract may be exposed to common carcinogens and the epithelium are at risk of malignant transformation either synchronously or metachronously of the index primary malignancy^1^. The development of second primary tumors (SPTs), especially in the head and neck, lung and esophagus regions, is not uncommon in head and neck squamous cell carcinoma (HNSCC) patients and the occurrence of SPTs is one of the leading causes of mortality^2^,^3^. The incidence of synchronous SPTs in HNSCC patients varies from 7% to 36%^4^,^5^,^6^,^7^,^8^,^9^. Additionally, the cumulative incidence of SPTs may be as high as 23.1% at 10 years and 36% at 20 years after the diagnosis of primary tumors^3^,^9^. Because the diversity of second cancers, it is still a challenge to manage the second cancers of different anatomic site in HNSCC patients.

The 5-year overall survival rate of esophageal cancer is less than 10~20% worldwide^10^. The most important reason for the poor prognosis is late diagnosis at advanced symptomatic stage. Among different sites of SPT in HNSCC patients, esophageal squamous cell neoplasia (ESCN) is the most common one involving the digestive tract^2^,^3^,^4^,^5^,^7^,^8^,^11^,^12^. Several studies have also demonstrated a worse prognosis for synchronous or metachronous ESCN in HNSCC patients^2^,^3^. Given that superficial ESCNs have lower chance for lymph node (LN) metastasis, whereas epithelial and lamina propria cancers have no risk for LN metastasis, and <10% of muscularis mucosa cancers and about 20% of superficial submucosa carcinomas have LN metastasis, the prognosis of patients with superficial ESCN is excellent, with 5-year survival rates over 80%^13^,^14^. Therefore, through endoscopic screening of esophagus in HNSCC patients, especially using image-enhanced endoscopy (IEE) technique, a substantial proportion of 3.2~28% ESCN could be identified before obstructive symptoms develop, and it was believed that with screening early ESCN before treatment of index primary tumor is associated with better outcome for HNSCC patients^15^,^16^.

At present, no standard treatments or guideline recommendations available to define whether index primary tumors or SPTs to be treated first. In addition, most of the previous reported screening ESCN in HNSCC patients are at precancerous or early cancerous stages, and about 15.5%~23.3% treatment strategy of HNSCC patients may be modified after detection of esophageal SPTs^4^,^7^,^8^,^15^,^16^. Although several retrospective studies reported routine IEE screening in newly diagnosed HNSCC patients could improve survival comparing to historic cohort^16^,^17^, without a prospective study, the long-term survival of these IEE identified HNSCC with ESCNs is still unclear. The aim of this study was to evaluate the prognosis of HNSCC patients with synchronous ESCN after IEE screening and to investigate the impacts of endoscopic screening on the treatment strategy and survival of index primary tumors.

## Materials and Methods

### Study Design

We prospectively recruited adults older than 20-year-old who had newly diagnosed head and neck cancers that were confirmed by two otolaryngology head and neck surgeons (L.-J. L., W.-C. L.) from March 2010 to April 2013 at the Far Eastern Memorial Hospital in New Taipei City, Taiwan. We excluded patients with salivary gland tumors, those who needed emergent surgery for compromised airways or tumor bleeding, trismus, allergic history to iodine and pregnant or those refused esophagogastroduodenoscopy (EGD) screening of esophagus. All enrolled patients were referred to gastroenterologists for EGD screening using IEE before treatment of index primary tumors. All the enrolled patients provided written informed consent before endoscopic examination. This study was approved by the Research Ethics Review Committee of Far Eastern Memorial Hospital (FEMH IRB-101022-E) and the methods were carried out in accordance with the relevant guidelines.

### Endoscopic screening of esophagus

**Figure 1:**
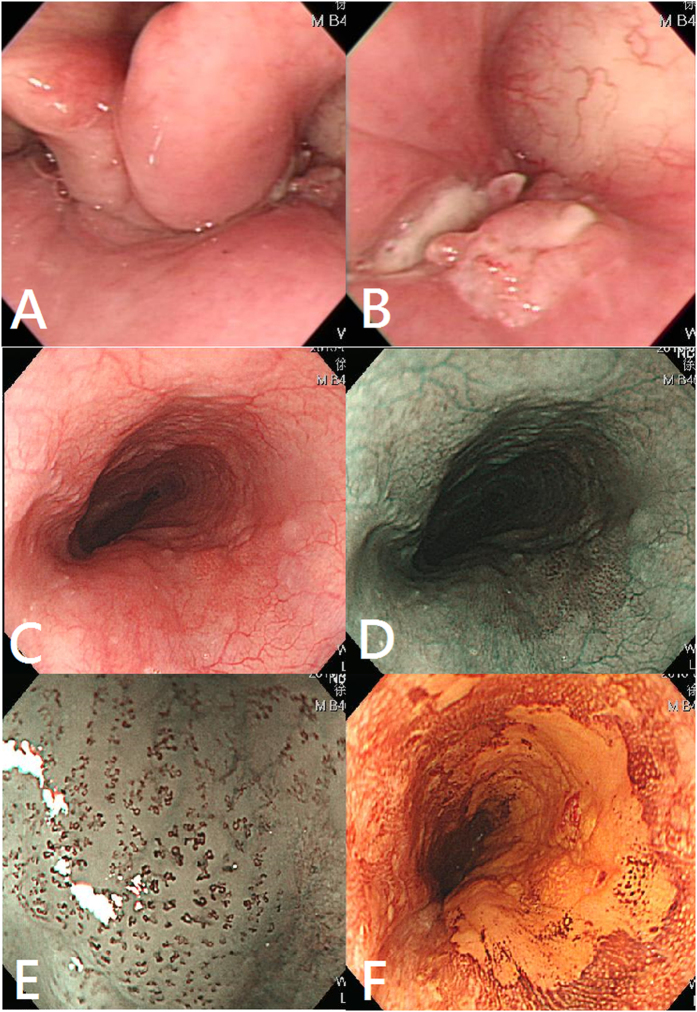
(**A**,**B)** A right-sided hypopharyngeal cancer. (**C**) An uneven hyperemic mucosa surface under white-light endoscopy. (**D**,**E**) Brownish discoloration of mucosa with abnormal microvasculatures under narrow-band imaging with magnifying endoscopy. (**F**) A well-demarcated Lugol-unstained area. Pathology from endoscopic submucosal dissection reporting squamous cell carcinoma invading the lamina propria.

All of the patients received endoscopic examinations using narrow-band imaging (NBI) with magnifying endoscopy (ME) which has powerful 80 times optical magnification (Evis Lucera CLV-260NBI, GIF-H260Z endoscopy, Olympus Medical Systems Corp, Tokyo, Japan), and chromoendoscopy with 2% Lugol’s solution (Sigma-Aldrich, St. Louis, Missouri, USA). NBI-ME and Lugol chromoendoscopy have been shown to identify second primary neoplasm in esophageal and head and neck cancer patients with pooled sensitivity and specificity of 87% and 95%, and 88% and 63%, respectively^18^. All endoscopic examinations and endoscopic submucosal dissection (ESD) of ESCN were performed by one experienced endoscopist (C.-S. C.) who had performed more than 250 EGDs equipped with NBI system and 4 ESDs monthly for 5 years. After an overnight fast, the patients swallowed a simethicone solution followed by local pharyngeal anesthesia with 10% xylocaine spraying. First, the esophagus was examined by conventional white-light imaging (WLI) endoscopy from the esophageal inlet to the esophagogastric junction, and then repeatedly evaluated backward under NBI-ME for esophagus. Secondly, after NBI examination, we switched back to WLI and sprayed 10 to 20 mL of 2% Lugol’s solution evenly over the mucosa from esophagogastric junction to upper esophagus. The definition of suspected ESCNs were defined as a hyperemic change, uneven or nodularity of mucosa under WLI system (Fig. 1C), or brownish discoloration of mucosa with abnormal intraepithelial capillary papillary loops (IPCLs) under NBI-ME system (Fig. 1D,E), or a well-demarcated Lugol-unstained area (Fig. 1F) with a diameter ≧5 mm or any Lugol-voiding lesions accompanied with pink color change 3 minutes after spraying Lugol’s solution which is often associated with high-grade neoplasia^4^,^19^,^20^. Finally, the stomach, the first and second portions of the duodenum, were examined under WLI endoscopy. Endoscopic biopsy was done for all suspicious lesions fulfilling the defined criteria.

### Histopathology and tumor board meeting for the treatment strategy

The biopsied tissues were examined by experienced pathologists and classified by the revised Vienna classification of epithelial neoplasia^21^. Negative for neoplasia/dysplasia was classified as category 1, chronic inflammation and squamous hyperplasia belonging to the diagnosis of indefinite for neoplasia were classified as category 2, non-invasive low-grade intraepithelial neoplasia (LGIN) was classified as category 3, high-grade intraepithelial neoplasia (HGIN) and carcinoma-*in-situ* were classified as category 4, and intramucosal or submucosa invasive carcinomas were category 5^21^. Patients with synchronous ESCNs with categories 1 and 2 received IEE survey with interval of 6-month, and those with categories 3~5 were considered to be managed^22^. The 7^th^ edition of the American Joint Committee on Cancer (AJCC) and the International Union for Cancer Control tumor-node-metastasis system was used for tumor staging^23^, and the treatment planning was made by tumor board review which was composed of expert opinions from gastroenterologists, radio-oncologists, surgical and medical oncologists. After a complete review of the medical condition of each patient and the information from loco-regional status, endoscopic and radiological examinations of primary tumors and SPTs, the final treatment options were made.

### Outcome Measurements and Statistical Analysis

We followed the survival condition of recruited patients. The over follow-up time was measured from initial diagnosis of primary cancer and till final death. If patients were lost during follow-up, they were classified as censors. The progression of dysplastic esophageal lesion was also followed by 6-month interval of surveillance EGD and recorded. Continuous data were expressed as mean (±standard deviation) or median (±interquartile range), category data were expressed as number (percentage). The overall survival of patients with/without SPTs of upper gastrointestinal (UGI) tract was plotted in Kaplan-Meier survival plots and Log-rank tests were used to compare overall survival (OS). Univariate and multivariate Cox-regression were used to test significant predictors for OS. All statistical analyses were accomplished using Stata software, version 12.0 (StataCorp LP, College Station, TX).

## Results

### Demographic data of study population

Totally two hundred and twenty-five HNSCC patients were diagnosed during the study period. One hundred and forty-five patients (66%) fulfilled the inclusion criteria and recruited in our study (Table 1). Their age was 56.1 (±9.8) years old and the female-to-male ratio was 6:94. The location of index primary tumors was oral cancer (63/43, no./%), oropharyngeal cancer (31/21), hypopharyngeal cancer (30/21), laryngeal cancer (16/11), and other HNSCCs (5/4). Among 80 head and neck cancer patients (demographic data shown in Supplementary Table 1) excluded from enrollment, five patients have allergic history to iodine, eight were salivary gland tumors, sixteen patients had trismus due to betel quid chewing, eighteen presenting tumor bleeding and impending airway compromised, and thirty-three refused IEE screening after informing the possible adverse effects from Lugol’s solution spraying. Study population was separated into two groups: synchronous ESCNs with revised Vienna classification of epithelial neoplasia categories 1~3 (group I), and categories 4~5 and other UGI tract malignancies (group II), with total number of 123 (84.8%) and 22 (15.2%) patients, respectively. The gender, age, clinical staging of index primary tumors, and status of habitual use of alcohol, cigarette, and betel quid were not significantly different between two groups. Higher proportion of hypopharyngeal cancers were noted in group 2 patients than those in group 1 (54% vs. 15%, p = 0.003). Higher proportion of alcohol drinkers was found in group 2 patients (86% vs. 68%, p = 0.085), but without statistically significance.

### Histopathology, stages and treatments of synchronous UGI neoplasia

A total of 67 biopsies of suspicious ESCNs and UGI tract neoplasia were carried out in 145 HNSCC patients. Regarding the histopathology, two, twenty-six (ten inflammation, sixteen squamonus hyperplasia), thirteen, eight and twelve biopsied esophageal samples were belonging to the revised Vienna classification category 1, 2, 3, 4, and 5, respectively. Two inadequate samples for histopathological diagnosis (probably esophagitis by IEE findings), two gastric adenocarcinomas, one esophageal subepithelial tumors and one Barrett’s esophagus were noted. Table 2 showed the characteristics of the HNSCC patients with synchronous SPTs of UGI tract, including 20 ESCNs (Vienna classification 4&5) and 2 gastric adenocarcinomas. Among those with synchronous ESCNs, 8 were HGIN (clinical stage 0), 6 were stage I (3 stage IA, 3 stage IB), 2 were stage II, 1 were stage IIIA and 3 were stage IV. Two were with synchronous gastric adenocarcinomas stage II and IV.

Treatments of SPTs were also shown in Table 2. Among HNSCC patients with synchronous esophageal HGIN, three patients (case 1, 4, 7) underwent ESD for esophageal lesions, four (case 2, 8, 9, 10) received endoscopic surveillance only because of short expected lifespan of primary tumors at advanced stages, poor nutrition and performance status, one (case 3) received concurrent chemoradiotherapy (CCRT) for both primary hypopharyngeal cancer and superficial ESCN. Among those with synchronous esophageal invasive carcinoma, one (case 19) underwent esophagectomy, two (case 5, 12) with esophagectomy and CCRT, six (case 6, 11, 13, 16, 17, 18) with CCRT only, and three patients (case 14, 15, 20) with initial predicted invasiveness of lamina propria by IEE underwent ESD with pathological reporting microinvasion of superficial submucosa layer. One patient (case 21) with gastric cancer underwent gastrectomy with chemotherapy and another one (case 22) received gastrectomy who developed metachronous ESCC at stage IIA four years later (loss follow-up for 2 years). Those with synchronous esophageal HGIN (case 2, 3, 8~10) without treatment remained histologically unchanged by surveillance IEE during study period.

### Overall Survival for HNSCC patients according to the existence of synchronous UGI neoplasia

**Figure 2:**
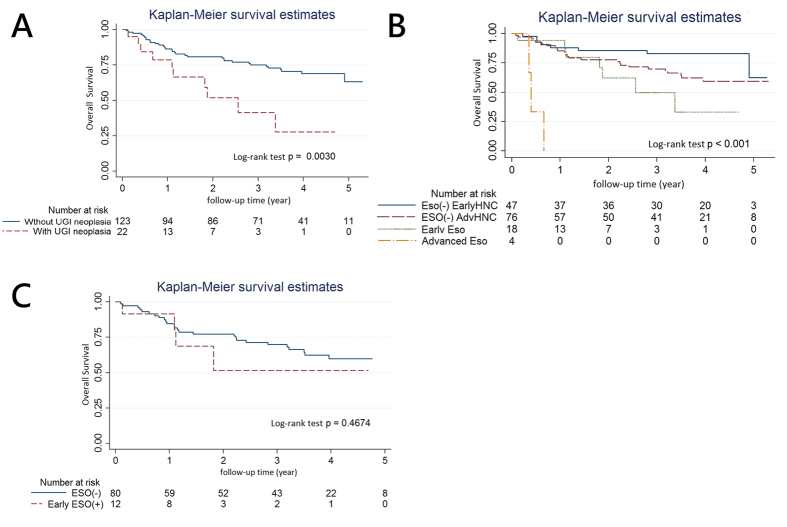
(**A**) Survival of head and neck squamous cell carcinoma (HNSCC) patients with synchronous upper gastrointestinal (UGI) tract neoplasia versus those without lesions. (**B**) Survival estimates according to staging of primary tumors and existence of synchronous esophageal neoplasia. (**C**) Survival estimates of advanced (stage III&IV) HNSCC patients with early esophageal neoplasia (stage 0~II) and those without lesions.

At a follow-up of 2.72 (±1.73) years, the median OS was 3.06 years (interquartile range ± 3.21 years). The overall 3-year survival rate was 0.71 (±0.04, 95% CI: 0.62~0.78). The OS rates of HNSCC patients according the existence of synchronous ESCNs and other UGI tract malignancies were shown in Fig. 2. When comparing the OS among the HNSCC patients who developed a SPT to those who did not, there was a statistically significant difference (Fig. 2A, p  = 0.003 Log-Rank test). HNSCC patients with advanced stages (III&IV) synchronous ESCN had worst survival rates (Fig. 2B, log-rank test p < 0.001). Using early HNSCC patients without synchronous ESCN as reference by cox regression analysis of survival, hazard ratios for advanced HNSCC patients without synchronous ESCN, those with synchronous early ESCN, and those with synchronous advanced ESCN were 2.02 (95% CI 0.9~4.55, p = 0.09), 3.70 (1.45~9.48, p = 0.01), and 39.36 (9.2~168.1, p < 0.01), respectively. Among advanced HNSCC patients, those without early ESCN, including HGIN and stage I&II esophageal cancers, had better survivals than those with synchronous lesions, but without statistical significance (Fig. 2C, p = 0.47). Cox regression analysis revealed that male gender, older age, advanced stages of primary tumors were associated with poorer outcomes, but without statistically significance (Table 3). Among those with synchronous ESCNs, hypopharyngeal cancers were associated with poorer prognosis when compared with oral cancers at univariate analysis (HR 2.36, 95% CI 1.08~5.15; p = 0.03). HNSCC patients with existence of esophageal/UGI tract neoplasia had poorer prognosis than those without, both at univariate (HR 2.92/2.85, 95% CI 1.37~6.22/1.38~5.87, both p < 0.01) and multivariate analyses (HR 2.75/2.79, 95% CI 1.11~6.82/1.15~6.80, p = 0.03/0.02).

## Discussion

The development of SPTs of UGI tract, especially esophagus, in HNSCC patients was not uncommon and usually associated with poor prognosis^2^,^3^,^24^. The incidence of synchronous SPTs outside head and neck region in HNSCC patients varies from 7~36%, and about 3.2~28% of HNSCC patients had synchronous esophageal neoplasia through IEE screening^4^,^5^,^6^,^7^,^8^,^9^,^15^,^16^. Carcinogenesis at multiple loci of squamous epithelium is common in HNSCC patients. Without routine endoscopy screening, the occurrence of synchronous or metachronous SPTs has been reported to be associated with poor prognosis. In a Turkish study of 1,112 HNSCC patients, Erkal *et al.* have demonstrated 7% and 9% patients presented with synchronous and metachronous neoplasia of head and neck mucosal sites, respectively^5^. The OS rate was 31% (*vs.* 45% for all enrolled patients) and the disease-specific survival rate was 50% (*vs.* 67%) at 5 years after diagnosis of metachronous HNSCC^5^. Notably, seven patients (1%) developed metachronous ESCN at 1 to 11.1 years (median, 2.8 years), and 83 patients (7%) developed metachronous lung cancers at 0.6 to 17.6 years (median, 3.5 years)^5^. Another long-term prospective study with a minimum follow-up of 10 years of 2,063 HNSCC patients showed an incidence of 17% for metachronous SPTs with median survival of 12 months only^25^. Moreover, the longer survival of HNSCC patients comes with higher incidence of SPTs. A Korean study of 937 HNSCC patients revealed the cumulative SPT incidence of 7.2% at 0 to 6 months (synchronous), 17.9% at 5 years, and 23.1% at 10 years after index primary tumor diagnosis^9^. These cohort studies without routine screening for SPTs highlighted the importance and impacts of SPTs in HNSCC patients.

HNSCC patients are at high risk for synchronous SPTs, especially neoplasia of UGI tract which is exposed to same environmental carcinogens as primary sites, illustrating the concept of “field cancerization”^1^,^2^,^3^. With the development of SPT of UGI tract in HNSCC patients, the prognosis is usually very poor despite advances in intensive multidisciplinary treatment strategy for HNSCC^2^,^3^,^5^,^9^,^25^,^26^. In the present study, we found that the incidence (15.2%) of SPT located at UGI tract using IEE screening in HNSCC patients was not low. HNSCC patients with synchronous esophageal/UGI tract neoplasia had poorer survival (HR 2.75/2.79, 95% CI 1.11~6.82/1.15~6.80, p = 0.03/0.02 in multivariate analyses). We also demonstrated that newly-diagnosed HNSCC patients with synchronous esophageal neoplasia at late stage (III&IV) had the worst outcome (Fig. 2B). The result shed lights on the importance of routine IEE screening of UGI tract neoplasia in newly-diagnosed HNSCC patients for risk stratification and prognosis prediction.

Recent advances in IEE imaging by means of dye-based or optical-based techniques have enabled precancerous or early cancerous lesions visible more easily than traditional WLI endoscopic examination^27^. Using IEE examination, especially chromoendoscopy with Lugol’s solution and NBI system with high-resolution ME, dysplastic or cancerous lesions, and tumor invasion could be well delineated and predicted^18^. Intentional IEE screening of esophagus in HNSCC patients identified higher proportion of SPTs (27.2% *vs.* 5.3%)^16^. Through IEE screening esophagus in HNSCC patients, a high incidence up to about 28% synchronous ESCN could be identified before obstructive symptoms develop^7^,^8^,^15^,^19^. Our previous experience has found 23.3% synchronous ESCN, including LGIN to invasive carcinoma in HNSCC patients and 15.5% treatment strategy of them had been modified after IEE screening for esophagus^8^. In this study, 15.2% HNSCC patients had advanced synchronous ESCN, including HGIN to invasive carcinoma, by IEE screening. Notably, the majority (Tables 2, 63.6% clinical stage 0 and I) of synchronous ESCN were at early stage without obstructive symptoms which made curative treatment of synchronous esophageal SPTs possible. However, poor nutrition and performance status of HNSCC with advanced ESCN hamper the concurrent treatment, which may lead to worst prognosis among these patients (Fig. 2B).

Due to the diversity of SPNs, the best management policy is still inconclusive. In this study, five patients presented early synchronous ESCN at stage 0 with stage III&IV primary index tumors received watch and wait or CCRT for SPTs, and none of them died of SPTs (Table 2). On the other hand, two esophageal SPTs at stage IB with primary index tumor at stage II and IV died of SPTs despite concurrent treatment of primary and secondary tumors. Among advanced HNSCC patients, although there was a trend for better survivals for those without early ESCN, the difference in survival was not statistically significant (Fig. 2C). Therefore, whether treatment of early SPTs in HNSCC patients with advanced stages improves their outcomes remains controversial and randomized studies with large sample size are needed to elucidate this issue.

Given that the occurrence of SPTs is associated with poor prognosis in HNSCC patients, identification of SPTs is considered to be beneficial for survival and cost-effective. A simulated Markov model-based analyses in an area with high risk for ESCN, China, has demonstrated that screening six times between 40–70 years at a 5-year interval yielded the highest net present value and benefit-cost ratio^28^. Thus, screening ESCN in high risk population including HNSCC patients should be cost-effective. A longitudinal cohort study compared the survival rates between HNSCC patients with and without routine IEE screening for ESCN^16^. The survival was better for those who received screening than those who did not (HR 0.57, 95% CI 0.41–0.79) and the Cox regression model quantified a survival benefit of 29% by IEE screening^16^. However, the real incidence of the occurrence of synchronous ESCN was unrevealed in HNSCC patient without IEE screening and lead-time bias existed in the retrospective study^16^. In our prospective study, every enrolled HNSCC patients underwent IEE screening for UGI tract neoplasia. The results have shown that the survival estimates were not influenced by the synchronous ESCN despite identification and treatment of SPTs, and as expected, the worst outcome was noted in HNSCC patients with advanced synchronous ESCN (Fig. 2B).

There were some limitations in our study. First, the sample size was small and follow-up period was short in this prospective cohort study conducted in one tertiary center. Large scale clinical trials in multicenter units with long time observation are needed to verify our results. Second, the treatment of identified SPTs was not standardized or randomized. However, it is difficult to conduct a randomized clinical trial to evaluate the impacts of treatment for early SPTs in those at advanced stage of index tumor based on individualized medicine approaching. The treatment strategy in this study was decided by tumor board conference and according to the status of nutrition, performance, location and staging of index and second primary malignancies. Additionally, underlying comorbidities were not included for survival analysis.

In conclusion, IEE screening could identify SPTs of UGI tract before symptoms develop and curative treatment of SPTs becomes possible by early endoscopic screening. The presence of SPTs was associated with poor prognosis, especially in those with advanced stage. By IEE screening, early prognostic stratification of HNSCC patients could be achieved. In this prospective design study which was different from previous similar study using historical controls^16^, survival benefits from IEE screening were not seen. However, further clinical trials are warranted to evaluate the benefits of treatment for early SPTs of UGI tract in advanced HNSCC patients.

## Additional Information

**How to cite this article**: Chung, C.-S. *et al.* Long Term Outcome of Routine Image-enhanced Endoscopy in Newly Diagnosed Head and Neck Cancer: a Prospective Study of 145 Patients. *Sci. Rep.* **6**, 29573; doi: 10.1038/srep29573 (2016).

## Supplementary Material

Supplementary Information

## Figures and Tables

**Table 1:** Demographic data of recruited patients.

Characteristics	N = 145	(%)	Group I (n = 123)	Group II (n = 22)	p-value
Sex (F/M)	8/137	6/94	6/117	2/20	0.425^β^
Age (years)	56.1 ± 9.8		55.57 ± 0.87	59.13 ± 2.15	0.117^@^
Primary site					0.003^#^
Oral cancer	63	43	58	5	
OP cancer	31	21	28	3	
Hypopharyngeal cancer	30	21	18	12	
Laryngeal cancer	16	11	14	2	
Other cancers	5	4	5	0	
Clinical-stage					0.850^#^
1	28	20	25	3	
2	21	15	18	3	
3	20	14	16	4	
4	71	51	60	11	
Alcohol drinking	103	71	84	19	0.085^#^
Betel quid chewing	91	63	80	11	0.179^#^
Cigarette Smoking	121	83	101	20	0.307^#^
Biopsy results (n = 67)					
Vienna classification					
1. Negative	2	3			
2. Inflammation	26	39			
3. LGIN	13	19			
4. HGIN	8	15			
5. Invasive Carcinoma	12	15			
6. Other*	6	9			

Group I: synchronous ESCNs with revised Vienna classification of epithelial neoplasia categories 1~3

Group II: synchronous ESCNs categories 4~5 and other UGI tract malignancies (group II).

LGIN: low-grade intraepithelial neoplasia; HGIN: high-grade intraepithelial neoplasia.

*Non-made: 2; Gastric cancer: 2; Esophageal submucosal tumor:1; Barrett’s esophagus: 1.

^β^Fisher’s exact test; ^@^t-test; ^#^Chi-square test.

**Table 2:** Patients with the diagnosis of esophageal cancer and high grade dysplasia 22/145 (15.2%).

Case	Primary tumor	cStage	SPT	cStage	Tx of SPT	Outcome
1	Larynx	I	EsoHGIN	0	ESD	LF
2	Hypo	IV	EsoHGIN	0	F/u	DH
3	Hypo	IV	EsoHGIN	0	CCRT	DH
4	Oropharynx	IV	EsoHGIN	0	ESD	DH
5	Hypo	III	ESCC	IIIA	OP+CCRT	DH
6	Oral	IV	ESCC	IB	CCRT	DE
7	Hypo	IV	EsoHGIN	0	ESD	DH
8	Hypo	III	EsoHGIN	0	F/u	NA
9	Oral	IV	EsoHGIN	0	F/u	DH
10	Oral	IV	EsoHGIN	0	F/u	NA
11	Hypo	IV	ESCC	II	CCRT	NA
12	Oropharynx	II	ESCC	IB	OP+CCRT	DE
13	Hypo	IV	ESCC	IV	CCRT	DH
14	Oral	II	ESCC	IA	ESD	DH
15	Hypo	III	ESCC	IA	ESD	LF
16	Oropharynx	IV	ESCC	IV	CCRT	NA
17	Hypo	III	ESCC	IV	CCRT	DE
18	Hypo	IV	ESCC	II	CCRT	LD
19	Larynx	I	ESCC	IB	OP	DH
20	Hypo	IV	ESCC	IA	ESD	LD
21	Oral	IV	GA	IV	OP+CT	DH
22^*^	Hypo	IV	GA	II	OP	NA

LF: Live without disease; LD: Live with disease; DH: Death due to HNSCC; DE: Death due to ESCN; F/u: follow-up; NA: Loss of F/u.

^*^Metachronous ESCC stage IIA 4 years later.

**Table 3:** Survival analysis with Cox Regression.

	Univariate Hazard ratio	95% CI	p-value	Multi-variate Hazard ratio	95% CI	p-value
Sex (M/F)	1.44/1.46	0.35~5.96/0.35~6.03	0.62/0.60	1.45/1.45	0.34~6.18/0.34~6.16	0.62/0.62
Age	1.00/1.01	0.97~1.04/0.98~1.05	0.79/0.46	0.99/1.01	0.96~1.03/0.97~1.04	0.74/0.78
Stage						
III&IV vs. I&II	1.94/1.97	0.98~3.87/0.99~3.90	0.06/0.05	1.81/1.87	0.85~3.84/0.88~3.95	0.12/0.10
Primary site						
Oral	1.00			1.00		
OP	1.27/1.19	0.57~2.82/0.54/2.63	0.56/0.66	1.12/1.01	0.49~2.55/0.45~2.27	0.79/0.99
Hypo	2.36/2.06	1.08~5.15/0.95~4.45	0.03/0.07	1.27/1.01	0.49~3.29/0.39~2.64	0.62/0.98
Larynx	1.21/1.14	0.44~3.35/0.41~3.12	0.71/0.81	1.10/0.99	0.39~3.12/0.35~2.78	0.86/0.98
Other	0.69/0.65	0.09~5.26/0.09~4.94	0.72/0.68	0.65/0.65	0.08~4.99/0.09~4.99	0.68/0.68
PES						
With SPT	2.92/2.85	1.37~6.22/1.38~5.87	<0.01/<0.01	2.75/2.79	1.11~6.82/1.15~6.80	0.03/0.02

Reported as esophageal neoplasia only/upper gastrointestinal tract neoplasia (including two gastric cancers).

## References

[b1] Slaughter D., Southwick H. & Smejkal W. Field cancerization” in oral stratifide squamous epithelium: clinical implications of multicentric origin. Cancer. 6, 963–968 (1953). 13094644 10.1002/1097-0142(195309)6:5<963::aid-cncr2820060515>3.0.co;2-q

[b2] Liao L. J., Chou H. W., Wang C. T., Chung C. S. & Lai M. S. The impact of second primary malignancies on head and neck cancer survivors: a nationwide cohort study. PLoS One. 8(4), e62116 (2013). 23614023 10.1371/journal.pone.0062116 PMC3628575

[b3] Morris L. G., Sikora A. G., Hayes R. B., Patel S. G. & Ganly I. Anatomic sites at elevated risk of second primary cancer after an index head and neck cancer. Cancer Causes Control 22(5), 671–679 (2011). 21327458 10.1007/s10552-011-9739-2 PMC3085084

[b4] Shiozaki H. *et al.* Endoscopic screening of early esophageal cancer with the Lugol dye method in patients with head and neck cancers. Cancer. 66(10), 2068–2071 (1990). 1699649 10.1002/1097-0142(19901115)66:10<2068::aid-cncr2820661005>3.0.co;2-w

[b5] Erkal H. S., Mendenhall W. M., Amdur R. J., Villaret D. B. & Stringer S. P. Synchronous and metachronous squamous cell carcinomas of the head and neck mucosal sites. J Clin Oncol. 19(5), 1358–1362 (2001). 11230479 10.1200/JCO.2001.19.5.1358

[b6] Muto M. *et al.* Association of multiple Lugol-voiding lesions with synchronous and metachronous esophageal squamous cell carcinoma in patients with head and neck cancer. Gastrointest Endosc. 56(4), 517–521 (2002). 12297767 10.1067/mge.2002.128104

[b7] Wang W. L. *et al.* Risk factors for developing synchronous esophageal neoplasia in patients with head and neck cancer. Head Neck. 33(1), 77–81 (2011). 20848418 10.1002/hed.21397

[b8] Chung C. S. *et al.* Risk factors for second primary neoplasia of esophagus in newly diagnosed head and neck cancer patients: a case-control study. BMC Gastroenterol. 13, 154 (2013). 24456340 10.1186/1471-230X-13-154 PMC4028981

[b9] Lee D. H. *et al.* Second cancer incidence, risk factor, and specific mortality in head and neck squamous cell carcinoma. Otolaryngol Head Neck Surg. 149(4), 579–586 (2013). 23820107 10.1177/0194599813496373

[b10] Stewart B. W. & Wild C. P. World Cancer Report 2014. Lyon 2014 (2014).

[b11] Graff P. *et al.* Management of patients with head and neck tumours presenting at diagnosis with a synchronous second cancer at another anatomic site. Clin Oncol (R Coll Radiol). 23(3), 174–181 (2011). 21130631 10.1016/j.clon.2010.10.008

[b12] León X. *et al.* Second primary tumors in head and neck cancer patients. Acta Otolaryngol. 122(7), 765–778 (2002). 12484655

[b13] Roth J. A. & Putnam J. B. J. Surgery for cancer of the esophagus. Semin Oncol. 21(4), 453–461 (1994). 7518967

[b14] Takubo K. *et al.* Early squamous cell carcinoma of the oesophagus: the Japanese viewpoint. Histopathology 51(6), 733–742 (2007). 17617215 10.1111/j.1365-2559.2007.02766.x

[b15] Chung C. S. *et al.* Secondary prevention of esophageal squamous cell carcinoma in areas where smoking, alcohol, and betel quid chewing are prevalent. J Formos Med Assoc. 109(6), 408–421 (2010). 20610142 10.1016/S0929-6646(10)60072-1

[b16] Wang W. L. *et al.* The benefit of pretreatment esophageal screening with image-enhanced endoscopy on the survival of patients with hypopharyngeal cancer. Oral Oncol. 49(8), 808–813 (2013). 23688774 10.1016/j.oraloncology.2013.04.009

[b17] Lim H. *et al.* Clinical significance of early detection of esophageal cancer in patients with head and neck cancer. Gut and liver. 9(2), 159–165 (2015). 25167869 10.5009/gnl13401 PMC4351021

[b18] Chung C. S. *et al.* Image-enhanced endoscopy for detection of second primary neoplasm in patients with esophageal and head and neck cancer: A systematic review and meta-analysis. Head Neck. Suppl 1, E2343–E2349 (2016). 26595056 10.1002/hed.24277

[b19] Muto M. *et al.* Early detection of superficial squamous cell carcinoma in the head and neck region and esophagus by narrow band imaging: a multicenter randomized controlled trial. J Clin Oncol. 28(9), 1566–1572 (2010). 20177025 10.1200/JCO.2009.25.4680 PMC2849774

[b20] Shimizu Y. *et al.* Endoscopic diagnosis of early squamous neoplasia of the esophagus with iodine staining: high-grade intra-epithelial neoplasia turns pink within a few minutes. J Gastroenterol Hepatol. 23(4), 546–550 (2008). 17573830 10.1111/j.1440-1746.2007.04990.x

[b21] Dixon M. F. Gastrointestinal epithelial neoplasia: Vienna revisited. Gut. 51(1), 130–131 (2002). 12077106 10.1136/gut.51.1.130 PMC1773259

[b22] Wang G. Q. *et al.* Histological precursors of oesophageal squamous cell carcinoma: results from a 13 year prospective follow up study in a high risk population. Gut. 54(2), 187–192 (2005). 15647178 10.1136/gut.2004.046631 PMC1774842

[b23] Edge S. B. & Compton C. C. The American Joint Committee on Cancer: the 7th edition of the AJCC cancer staging manual and the future of TNM. Ann Surg Oncol. 17(6), 1471–1474 (2010). 20180029 10.1245/s10434-010-0985-4

[b24] Chen M. C., Huang W. C., Chan C. H., Chen P. T. & Lee K. D. Impact of second primary esophageal or lung cancer on survival of patients with head and neck cancer. Oral Oncol. 46(4), 249–254 (2010). 20138797 10.1016/j.oraloncology.2010.01.002

[b25] Rennemo E., Zätterström U. & Boysen M. Impact of second primary tumors on survival in head and neck cancer: an analysis of 2,063 cases. Laryngoscope. 118(8), 1350–1356 (2008). 18496157 10.1097/MLG.0b013e318172ef9a

[b26] Griffioen G. H. *et al.* Second primary lung cancers following a diagnosis of primary head and neck cancer. Lung Cancer 88(1), 94–9 (2015). 25662386 10.1016/j.lungcan.2015.01.011

[b27] Kaltenbach T., Sano Y., Friedland S., Soetikno R. & Association A. G. American Gastroenterological Association (AGA) Institute technology assessment on image-enhanced endoscopy. Gastroenterology. 134(1), 327–340 (2008). 18061178 10.1053/j.gastro.2007.10.062

[b28] Yang J. *et al.* Cost-benefit analysis of esophageal cancer endoscopic screening in high-risk areas of China. World J Gastroenterol. 18(20), 2493–2501 (2013). 22654446 10.3748/wjg.v18.i20.2493 PMC3360447

