# Supplementary material for: Long Term Outcome of Routine Image-enhanced Endoscopy in Newly Diagnosed Head and Neck Cancer: a Prospective Study of 145 Patients
Source: Sci Rep. 2016 Jul 8;6:29573. doi: 10.1038/srep29573 (PMC4937417; doi:10.1038/srep29573)
Supplement: Supplementary Information [file srep29573-s1.doc]

**Long Term Outcome of Routine Image-enhanced Endoscopy in Newly Diagnosed Head and Neck Cancer: a Prospective Study of 145 Patients**

Chen-Shuan Chung,^1,2^ MD; Wu-Chia Lo^3^, MD ; Ming-Hsun Wen,^3^ MD; Chen-Hsi Hsieh,^4,5^ MD, PhD; Yu-Chin Lin,^6^ MD, PhD; Li-Jen Liao,^3,7^ MD, PhD

Departments of Internal Medicine^1^, Otolaryngology^3^, Radiation Oncology^4^, Medical Oncology and Hematology^6^, Far Eastern Memorial Hospital, New Taipei City, Taiwan

^2^College of Medicine, Fu Jen Catholic University, New Taipei City, Taiwan

^5^Department of Medicine, School of Medicine, National Yang-Ming University, Taipei, Taiwan

^7^Department of Electrical Engineering, Yuan Ze University, Taoyuan, Taiwan

**Supplementary table 1. Demographic data of recruited and excluded patients**

|  |  |  |  |  |
| --- | --- | --- | --- | --- |
| Characteristics | Enrolled patients n=145 (%) | Excluded patients n=80 (%) | p-value | Total  225 |
| Sex(F/M) | 8 (5.52) / 137 (94.48) | 11 (13.75) / 69 (86.25) | 0.034 | 19/206 |
| Age | 56.1±9.8 | 55.8±11.2 | 0.515 | 55.8±10.3 |
| Primary site |  |  | 0.001 |  |
| Oral cancer | 63 (43.45) | 45 (56.25) |  | 108 |
| Oropharyngeal cancer | 31 (21.38) | 12 (15.0) |  | 43 |
| Hypopharyngeal cancer | 30 (20.69) | 14 (17.5) |  | 44 |
| Larynx cancer | 16 (11.03) | 0 (0) |  | 16 |
| Other cancers | 5 (3.45) | 9 (11.25) |  | 14 |
| Clinical-stage |  |  | 0.340 |  |
| 1 | 28 (19.31) | 12 (15.00) |  | 40 |
| 2 | 21 (14.48) | 19 (23.75) |  | 40 |
| 3 | 20 (13.79) | 11 (13.75) |  | 31 |
| 4 | 76 (52.42) | 38 (47.50) |  | 114 |
